# Multiplex PCR as a tool for the diagnosis of *Leishmania* spp. kDNA and the gapdh housekeeping gene of mammal hosts

**DOI:** 10.1371/journal.pone.0173922

**Published:** 2017-03-16

**Authors:** Renata de Cássia-Pires, Myllena de Fátima Alheiros Dias de Melo, Raquel da Hora Barbosa, André Luiz Rodrigues Roque

**Affiliations:** 1 Laboratory of Trypanosomatid Biology, Oswaldo Cruz Institute, FIOCRUZ, Rio de Janeiro, Rio de Janeiro, Brazil; 2 Laboratory of Molecular Biology and Endemic Diseases, Oswaldo Cruz Institute, FIOCRUZ, Rio de Janeiro, Rio de Janeiro, Brazil; 3 Center of Medical Sciences, Biomedical Institute, Fluminense Federal University, UFF, Niterói, Rio de Janeiro, Brazil; Academic Medical Centre, NETHERLANDS

## Abstract

**Background:**

The PCR assays usually employed for *Leishmania* diagnosis does not simultaneously detect a constitutive gene that would certify the viability of the DNA sample. We present a multiplex PCR approach for the simultaneous diagnosis of the *Leishmania* sp. kDNA fragment and a catalytic domain segment of a conserved region of the mammalian gapdh gene.

**Methodology:**

The proposed multiplex protocol was designed through *in silico* PCR. The annealing temperature, concentration of primer pairs, number of cycles, distinct polymerase enzymes and premix kit were defined to achieve an optimal reaction. The DNA detection sensitivity was tested with different concentrations of known *L*. *tropica* DNA, and the reproducibility of the assay was confirmed using samples from 106 wild mammals that were previously subject to *Leishmania* sp. kDNA analysis through singleplex reactions.

**Principal findings:**

The following optimal conditions were established: 95°C for 1 min followed by 30 cycles of 95°C for 30 s, 61°C for 30 s, and 72°C for 30 s and a final extension at 72°C for 1 min. The multiplex PCR system was capable of detecting 0.1 ng of *L*. *tropica* diluted in 100 ng of mammalian DNA. Of 51 kDNA samples that were previously found to be positive, 45 (88.2%) were positive for both targets, two were positive only for kDNA and four were negative for both. Of 55 kDNA samples that were previously identified as negative, 38 (69.1%) were positive for gapdh whereas the other 17 were negative for both targets.

**Conclusions/Significance:**

The proposed multiplex PCR system allows the simultaneous detection of the gapdh gene and *Leishmania* sp. kDNA in tissue samples derived from distinct wild mammal species. The amplification of the gapdh mammalian gene in the same reaction ensures the quality and viability of the DNA in the sample, eliminating the possibility of false-negative results that would impair an accurate description of the infection rates in a given population.

## Introduction

Leishmaniases are a group of parasitic diseases caused by heteroxenous protozoans from the *Leishmania* genus (Ross 1903). This genus is composed of approximately 30 species, approximately 20 of which are described as pathogenic to humans [1]. *Leishmania* species are involved in complex transmission cycles that include domestic and/or wild mammals from nine different orders and dozens of insect vector species from the Phlebotominae family [2–3].

To overcome the limitations of the traditional parasitological methods (related to the difficulties associated with the isolation of parasites, usually due to a low parasite load and an irregular distribution among host tissues), molecular approaches aiming to detect *Leishmania* species directly in biological samples have been developed. The advantage of molecular approaches based on Polymerase Chain Reactions (PCRs) is the combination of high sensitivity for parasite detection in various human, animal and sand fly tissues with target specificity [4–5]. Additionally, the possibility of combining multiple targets in the same assay enables the identification of parasites and the evaluation of sample integrity and PCR performance in the diagnosis of infection from Phlebotominae sandflies in some studies [6–7]. The same approach, although proposed for mammals [8–9], is rarely employed because these studies sought to investigate infection by specific *Leishmania* species: namely *L*. *braziliensis* and *L*. *infantum*.

The kDNA is the most employed molecular target in the routine diagnosis of *Leishmania* infection because of its recognized high sensitivity and specificity for the detection of infection, and the use of this target allows parasite identification at the genus or, at most, subgenus level [10]. The genes responsible for expression of the glyceraldehyde-3-phosphate dehydrogenase (gapdh or G3PD) enzyme are present in all mammalian cells [11]. Thus, the detection of gapdh is a potential tool for controlling the quality of samples of mammalian tissues used for PCR In the present study, we developed a multiplex PCR system that allows direct diagnosis from tissue fragments of different mammalian hosts by targeting a region of kinetoplast (kDNA) conserved in several species of the *Leishmania* genus and a constitutive gene (gapdh) of the mammal hosts.

## Materials and methods

### Ethics statement

All of the procedures carried out with these animals were authorized by the Brazilian Institute of Environment and Renewable Natural Resources (IBAMA) and followed protocols approved by the Ethics Committee of Animal Use of Fiocruz (P0007-99, P0179-03, P0292/06, and L0015-07). The non-infected human DNA used as a PCR control was kindly donated by Dr. Constança Britto and derived from studies approved by the Ethics Committee and conducted in the Laboratory of Molecular Biology and Endemic Diseases (IOC/Fiocruz).

### Primer design and *in silico* analysis

A conserved region in the gapdh gene was used to design a primer pair that is able to detect DNA from several mammalian species, including wild animals. Considering the size of the *Leishmania* sp. amplicon in kDNA reactions [120 base pairs (bp)], we aimed to obtain a mammalian conserved region to be in close proximity, and identified a region that could yield amplicons with 212 bp. Representative gapdh mammalian genomes (*Homo sapiens*—NM_001289745.1; *Pan troglodytes*—XM_508955.6; *Mus musculus*—NM_001289726.1; *Rattus norvegicus*—NM_017008.4; *Monodelphis domestica*—XM_007503905.1; *Equus caballus*—NM_001163856.1; *Felis catus—*NM_001009307.1; *Bos taurus*—NM_001034034.2; *Myotis lucifugus*—XM_006084148.2); *Leishmania* spp. genomes (*L*. *donovani—*13388249; *L*. *infantum—*5073932; and *L*. *mexicana–* 13448275; and glycosomal gene sequences identified in various *Leishmania* species (*L*. *major–* 5653844; *L*. *braziliensis*– 5417820; *L*. *tarentolae*—DQ092549; *L*. *panamensis* -22577345; *L*. *infantum*—KF041811; and *L*. *lainsoni*—KP197180) were retrieved from the GenBank database and used in FASTA format to identify the conserved region. The sequences were aligned using CLC Workbench v.6 software (Qiagen, Valencia, CA, USA). The gapdh primer pair was designed with Primer3 software [12].

The phylogenetic relationship of the target gapdh sequence of mammals and *Leishmania* species was inferred using a Neighbor Joining (NJ) tree with 1,000 replicates that was constructed based on the Kimura 80 distance measure using the CLC-Main Workbench software 6.9.1 (Bio, Qiagen, Denmark). To infer similarity and minimize nonspecific detection, the oligonucleotides were compared with gapdh genes using the BLAST database search program (provided online by the National Center for Biotechnology Information—NCBI www.ncbi.nml.nih.gov). An *in silico* analysis, which was conducted using the Multiple Primer Analyzer web tool from ThermoScientific^™^ (www.thermoscientific.com/webtools/multipleprimer) was also performed to evaluate the possibility of using the oligonucleotides in the multiplex reaction and dimer detection. The second primer pair used to amplify sequences of the *Leishmania* spp. kDNA conserved region was previously described [10] (Table 1).

**Table 1:** DNA sequence and genomic region of molecular targets employed in the multiplex PCR system.

Organism	Primer sense	Primer antisense	Genomic region	Fragment size (bp)	References
*Leishmania* sp. kDNA	5’GGG(G/T)AGGGGCGTTCT(C/G)CGAA-3’	5’(C/G)(C/G)(C/G)(A/T)CTAT(A/T)TTACACCAACCCC 3’	Mitochondrial, chromosome 26	120	Degrave et al., 1994
Vertebrate host DNA	5`ACCACAGTCCATGCCATCAC 3’	5`GTCAGGTCCACCACTGACAC 3’	Nuclear, chromosome 12	212	This study

### DNA of control samples

Positive DNAs were obtained from spleen and liver fragments of hamsters infected with *L*. *braziliensis* (MHOM/BR/2000/LTCP13396 = IOC-L2483) [13]. DNA of samples from *Leishmania tropica* (MHOM/AZ/1958/STRAINOD) and *L*. *tarentolae* (LEXSinduce4 Expression Kit, Jena Bioscience, Jena, GE) were also used as positive controls. All parasites were derived from Fiocruz’ Institutional Collections: Coleção de *Leishmania* do Instituto Oswaldo Cruz (CLIOC) and Coleção de Protozoários (COLPROT). Negative controls were obtained from spleen and liver fragments of non-infected hamsters and non-infected DNA extracted from *Homo sapiens* blood and nail samples. Blood and nail samples were extracted using a QIAamp DNA Mini kit (Qiagen, Valencia, CA, USA). Tissue fragments and promastigote forms of *Leishmania* were extracted using a Wizard Genomic DNA Purification Kit (Promega, Madison, WI, USA). All procedures were performed according to the manufacturer’s recommendations.

### Multiplex PCR standardization

All PCR standardizations were performed in 0.2-ml plastic tubes in a Mastercycler Nexus Thermal Cycler (Eppendorf^™^, Hamburg, Germany) using the DNA control samples mentioned above. A premix volume sufficient for the number of reactions was prepared, and this premix consisted of 2 μL of DNA template in a final volume of 25 μL.

PCR standardization was initiated with a touchdown PCR protocol [14] to verify the ideal conditions for amplifying the gapdh target fragment. Gradient PCR with temperatures ranging from 57°C to 61°C was performed using both pairs of primers (kDNA and gapdh). The PCR mix contained 10X buffer, 0.4 mM of each deoxyribonucleoside triphosphate (dNTPs), 1.25 U of Taq DNA polymerase (G008, abm^®^) and 10 μM of each primer.

Using an annealing temperature of 61°C and maintaining the remaining reagent concentrations, we tested different concentrations of each primer pair (ranging from 5 to 25 μM) in the same reaction. The reactions were performed using the following conditions: 95°C for 1 min followed by 40 cycles of 95°C for 30 s, 61°C for 30 s, and 72°C for 30 s and a final extension at 72°C for 1 min. These tests were also performed with and without the addition of 5% dimethyl sulfoxide (DMSO).

With the optimal concentrations kDNA and gapdh primers of 15 and 5 μM, respectively, the amplification efficiency was tested using the Taq DNA polymerase enzymes (ABM^®^) and FideliTaq PCR Master Mix (Affymetrix, USB). In both of these tests, we reduced the number of cycles from 40 to 30 without changing the remaining conditions. Finally, we compared the success of the multiplex PCRs with that of the reactions using premix Ready-To-Go PCR beads (Amersham Biosciences, Buckinghamshire, UK).

### Sensitivity test

The sensitivity of DNA detection by multiplex PCR was defined by employing DNA from *L*. *tarentolae*, *L*. *tropica* and *H*. *sapiens* using mixtures consisting of a constant concentration of human DNA (100 ng) and different concentrations of *L*. *tropica* DNA (100, 50, 10, 5, 1, and 0.1 ng). The *L*. *tarentolae* DNA was used to control the specificity of the gapdh gene.

### Multiplex PCR reactions with samples from wild mammals

After establishing the multiplex PCR system using the control DNA samples, we tested its reproducibility using samples of different tissues from 106 wild mammals (Table 2). All of these samples had been previously tested for *Leishmania* sp. kDNA in singleplex reactions using the same primers and conditions in a previous study [13], and the results revealed that 51 and 55 of these samples were positive and negative, respectively.

**Table 2:** Details of the samples employed to validate the multiplex PCR system: Mammal order, geographic origin and tissue from wild mammalian hosts previously diagnosed with *Leishmania* spp. infection using a singleplex reaction.

Mammal Order	Federal Unit	Tissue Fragment	kDNA (+)	kDNA (-)
Chiroptera	Paraíba	Li (7); Sk (9); Spl (16)	Li (7); Sk (7); Spl (14)	Sk (2); Spl (2)
	Rio de Janeiro	Spl (2)	Spl (1)	Spl (1)
	Santa Catarina	Spl (2)	0	Spl (2)
Didelphimorphia	Bahia	Spl (1)	Spl (1)	0
	Goiás	Li (8); Sk (4)	Li (1)	Li (7); Sk (4)
	Paraíba	Li (1); Sk (1); Spl (18)	0	Li (1); Sk (1); Spl (18)
	Rio de Janeiro	Spl (1)	0	Spl (1)
Lagomorpha	Paraíba	Li (1); Sk (1)	Li (1); Sk (1)	0
Rodentia	Bahia	Spl (2)	Spl (2)	0
	Goiás	Li (6); Sk (8)	Li (1)	Li (5); Sk (8)
	Mato Grosso do Sul	Spl (10)	Spl (8)	Spl (2)
	Paraíba	Li (1)	0	Li (1)
	Piauí	Spl (7)	Spl (7)	0
		**N = 106**	**N = 51**	**N = 55**

Li: Liver; Sk: Skin; Spl: Spleen

### Gel electrophoresis and documentation

The PCR products were visualized after electrophoresis on an 8% polyacrylamide gel and subjected to silver staining using a kit (DNA Silver Staining, GE Healthcare). A 50-bp DNA ladder (Promega, Madison, WI, USA) was employed as the base pair length marker, and gel pictures were taken using a GS-800^™^ Calibrated Densitometer (Bio-Rad, CA, USA).

## Results

**Fig 1:**
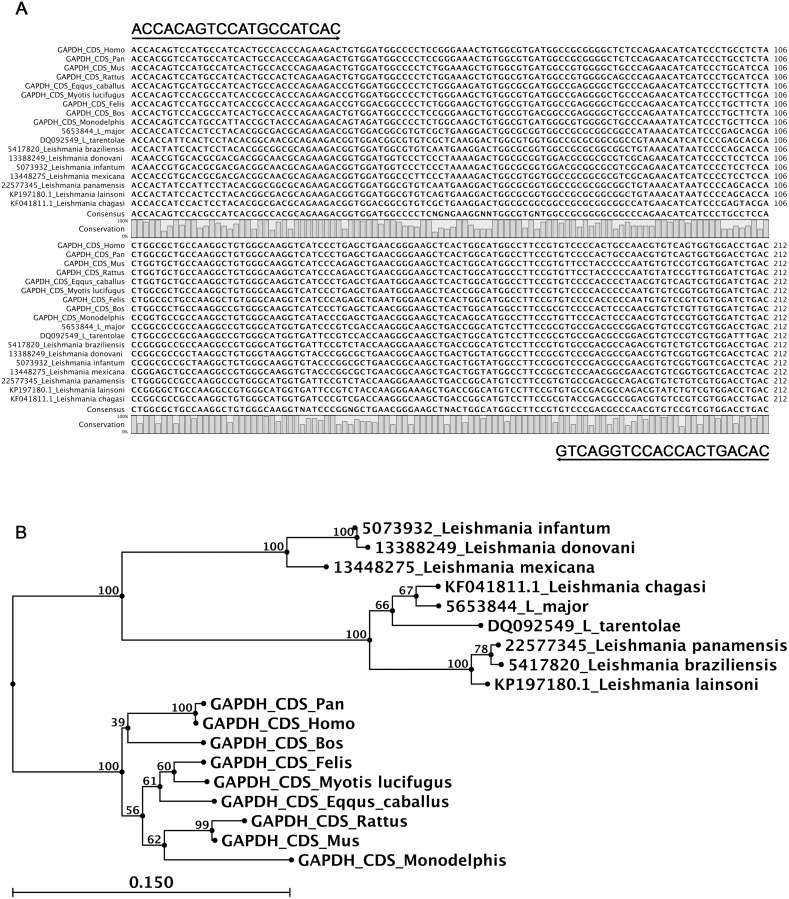
gapdh alignment among mammal and *Leishmania* species and phylogenetic tree of the gapdh sequences. (A) Multiple alignment of the target gapdh segment (212 bp) of different mammals and *Leishmania* species using ClustalW in CLC-Main Workbench software (6.9.1). All sequences were accessed from the NCBI and EMBL-EBI databases using UniProt and InterPro resources. The alignment showed 122 (57%) nucleotides that were conserved in different species, whereas 167 (79%) were conserved in mammal sequences (variable height in the bar plot). (B) A phylogenetic tree of the target gapdh sequence (212 bp) of different mammal and *Leishmania* species based on the neighbor joining method showed two divergent branches: mammals and Leishmania clusters. Arrows—localization and orientation of each primer in the alignment; bar plot—nucleotide conservation; consensus—sequence generated through the most conserved nucleotides between all aligned sequences; tree parameters: neighbor joining tree with 1,000 replicates and constructed based on the Kimura 80 distance measure using CLC-Main Workbench software (6.9.1).

The analysis of the genetic similarity among gapdh genes emphasized the importance of the coding sequence (CDS) segment and the catalytic domain. This gene, which is present as a single copy and has been mapped to chromosome 12 in the *Homo sapiens* genome [15], showed a high density of sequences that were conserved among its orthologous genes (S1 Fig). Using the information obtained from the alignment of the mammalian gapdh sequences, two candidate oligonucleotide primers were proposed for amplifying a segment of approximately 212 bp from the catalytic domain. The alignment of the target gapdh sequence (212 bp) showed that 122 (57%) nucleotides were conserved in different species (bar plot height variation, Fig 1A), and 167 (79%) nucleotides were conserved in mammal sequences (Fig 1A). The target gapdh sequence (212 bp) of mammal and *Leishmania* species diverged into two clearly separate branches, as illustrated in the phylogenetic tree (Fig 1B). In addition, despite an *in silico* analysis showed a high sequence identity (more than 90%) between the gapdh oligonucleotides and the representative of gapdh sequences (S1 Table), the results of total score, coverage and e-value using *Leishmania* gapdh sequences were lower than mammal gapdh data, which suggested low probability of hybridization with different *Leishmania* DNA samples. These oligonucleotides can be combined with the *Leishmania* spp. kDNA in a multiplex assay because no specificity is lost by alignment with their specific targets, resulting in a lack of dimer detection.

The temperature was the first aspect of the multiplex reaction that we standardized, and the results revealed a temperature of 61°C was demonstrated to be more effective for annealing both pairs of primers with their respective target sequences. Nevertheless, the PCR conditions established at this temperature and with 40 cycles sometimes resulted in some non-specific bands (approximately 400 bp), which are heavier than those of the targets of interest. The addition of 5% DMSO eliminated almost all non-specific bands.

**Fig 2:**
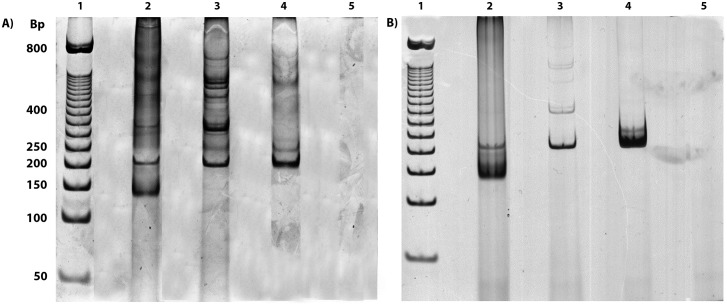
Silver-stained electrophoresis polyacrylamide gel obtained from the amplification of gapdh and kDNA with the multiplex PCR system. (A) PCR products from the multiplex PCR system using Taq DNA polymerase enzymes (ABM^®^) in the presence of 5% DMSO. (B) PCR products from the multiplex PCR system using the FideliTaq PCR Master Mix (Affymetrix, USB). In both figures: 1. molecular-weight marker (50-bp DNA ladder); 2. infected rodent; 3. uninfected rodent; 4. human DNA; and 5. negative PCR control.

**Fig 3:**
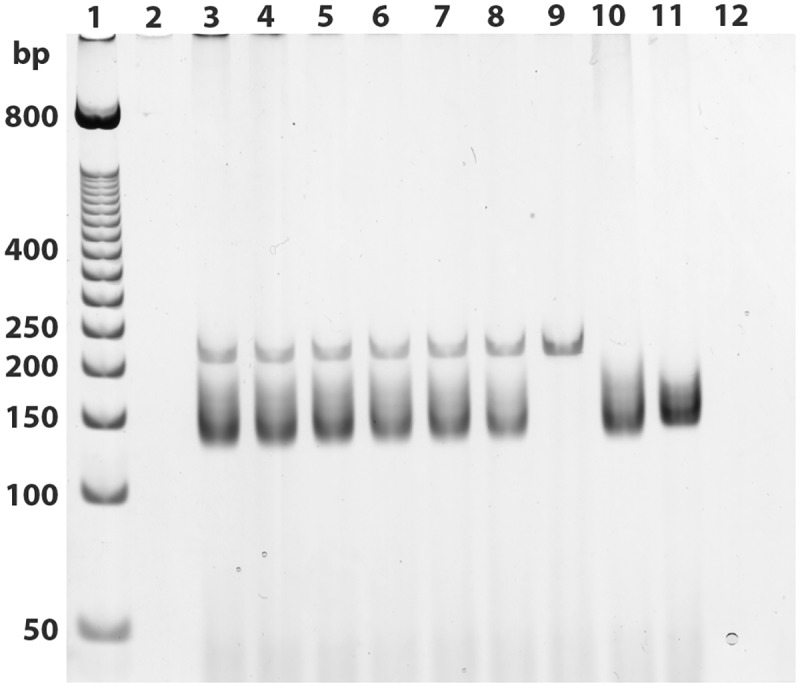
Silver-stained electrophoresis polyacrylamide gel obtained from the sensitivity test for detecting *Leishmania* sp. DNA with a constant concentration of human DNA (100 ng). 1: molecular-weight marker (50-bp DNA ladder); 2. empty; 3–8. mixture of human DNA and 100 (3), 50 (4), 10 (5), 5 (6), 1 (7) and 0.1 (8) ng of *L*. *tropica*; 9. uninfected rodent; 10. *Leishmania tropica* DNA; 11. *Leishmania tarentolae* DNA; and 12. Negative control for the PCR.

After testing different concentrations of each primer, the best optimal conditions for amplification of both primers were identified using concentrations of the gapdh target and the kDNA equal to 5 and 15 μM, respectively. The reactions were successfully employed using two different Taq enzymes; however, the use of the Taq DNA polymerase enzyme (ABM^®^) showed some unspecific heavier than the fragments of interest, whereas a slightly better resolution was obtained using the FideliTaq PCR Master Mix (Affymetrix, USB) with a cycling profile of only 30 cycles (Fig 2). Once the conditions and cycling profile of the multiplex PCR assay were established, the reactions were also tested using Premix PuReTaq Ready-To-Go PCR beads, and the results were similar to those obtained from the amplifications performed with the FideliTaq PCR Master Mix (Affymetrix, USB). We were able to detect up to 0.1 ng of *Leishmania* sp. DNA from *L*. *tropica* diluted in 100 ng of mammalian DNA. Only the corresponding gapdh and kDNA bands were observed in human and *Leishmania* samples, respectively (Fig 3).

One hundred six wild mammalian samples were tested using the PuReTaq Ready-To-Go PCR beads (Amersham Biosciences, Buckinghamshire, UK) with the following conditions: 95°C for 1 min followed by 30 cycles of 95°C for 30 s, 61°C for 30 s, and 72°C for 30 s, and a final extension at 72°C for 1 min. Of 51 samples that were previously found to be positive for the kDNA target through singleplex reactions, 45 (88.3%) amplified both pair of primers, as expected. Two liver samples (one from a marsupial and one from a rodent) were positive only for the kDNA target. The four remaining samples (three derived from bat’s skin and one obtained from a fragment of rodent spleen) were negative for both primer sets, and this result was likely due to DNA degradation after the singleplex reaction, which had been performed four years before. Of the 55 samples that were previously found to be negative for the kDNA target, all yielded negative results in the multiplex reactions, but only 38 of these (69.1%) tested positive for the gapdh control, suggesting that the remaining 17 samples had degraded, and are thus prone to yield false-negative results (Table 3). The 21 mammal samples (17 and four that were previously considered positive and negative, respectively) that were found to be negative for both targets using the multiplex PCR were tested again using through a singleplex PCR for *Leishmania* sp. kDNA and this re-testing yielded negative results.

**Table 3:** Amplification of the molecular targets used in the multiplex PCR system using samples from wild mammalian hosts.

Order	Tissue	(+) for both targets	(-) for kDNA and (+) for gapdh	(-) for both targets	(+) for kDNA and (-) for gapdh
Chiroptera	Li	7	0	0	0
	Sk	4	2	3	0
	Spl	15	5	0	0
Didelphimorphia	Li	0	8	0	1
	Sk	0	5	0	0
	Spl	1	15	4	0
Lagomorpha	Li	1	0	0	0
	Sk	1	0	0	0
	Spl	0	0	0	0
Rodentia	Li	0	0	6	1
	Sk	0	1	7	0
	Spl	16	2	1	0
		**N = 45**	**N = 38**	**N = 21**	**N = 2**

## Discussion

Molecular tools for the diagnosis of *Leishmania* infections are routinely employed for humans, vectors, and wild and domestic mammalian samples [6,13,16–17]. This intracellular parasite is heterogeneously distributed in mammal tissues and is usually described in low parasite loads, at least in wild mammals [2]. For this reason, targets directed to the conserved region of *Leishmania* kDNA are the most employed due to their high sensitivity of detection, which is a result of the estimated 10–20 thousand mini-circle copies of kDNA in each parasite cell [18]. Despite the high sensitivity of this molecular target, false-negative results could be related to inappropriate DNA extraction and/or DNA degradation after extraction, and these false-negative results impact the calculation of infection rates, resulting in underestimation and sometimes minimization of a serious public health problem. Based on the high sensitivity of the kDNA target, we proposed a molecular test that maintains the detection sensitivity for *Leishmania* sp. DNA while simultaneously evaluating the sample integrity by detecting a constitutive mammalian gene as an internal reaction control.

Many housekeeping genes (such as the mammal gapdh gene) can be used as an internal control for sample viability in molecular assays [19–20]. Here, we designed new oligonucleotides capable of amplifying smaller fragments of the gapdh gene that can be used in combination with a primer pair targeting *Leishmania* kDNA without affecting the PCR conditions. These proposed oligonucleotides amplified a highly conserved region of the catalytic domain, with a degree of specificity that enables its application to different mammalian samples that show more than 90% identity among sequences from the GenBank database and no cross-reaction with *Leishmania* genomes was noted.

Despite the excellent sensitivity of the Fidelity Taq^®^ enzyme, their use in our standardization showed no significant differences between this enzyme and the common Taq DNA enzymes. The use of PCR premix beads (PuReTaq Ready-To-Go PCR beads^®^) allows greater reproducibility and lessens the chance of contaminating the reactions due to decreased reagent handling and higher control over the concentrations of each reagent employed [13,21]. As determined through dilution of the known concentrations of *Leishmania* sp. DNA, we were able to amplify 0.1 ng of *Leishmania* diluted in 100 ng of mammalian DNA with high efficiency. The detection limit of these tests, in addition to the results obtained from the validation test with wild mammalian samples, showed that the multiplex PCR system proposed herein can be universally employed for the diagnosis of *Leishmania* infection in mammalian tissues, without any loss of efficiency.

To the best of our knowledge, only two published manuscripts present multiplex PCR tools for the diagnosis of *Leishmania* infections and a concomitantly evaluation of sample viability through the detection of the mammalian gapdh sequence. One of these studies focused on *L*. *braziliensis* [9], and the other investigated both *L*. *braziliensis* and *L*. *infantum* [8]. In contrast, the multiplex system described in this manuscript is able to detect any species within the *Leishmania* genus direct in tissues, independent of symptoms or suspicion of infection by one or other *Leishmania* species. The *Leishmania* genus comprises more than 30 species [1], and almost all of these circulate in the wild in complex cycles maintained by several potential reservoir species [2]. The *Leishmania* species that infect wild mammals or vectors in the wild might be quite different from the currently accepted distribution map of *Leishmania* species, which is based only on human and/or canine infection [13,22]. The herein proposed multiplex system can be applied for the identification of other *Leishmania* species rather than only *L*. *braziliensis* or *L*. *infantum* in any wild mammal host, independent of the geographic region or local epidemiological characteristics.

In addition, the multiplex system presented herein displayed high applicability for the detection of *Leishmania* sp. infection, regardless of the tissue fragment and/or mammal host. Four samples that were previously found to be positive for kDNA through singleplex PCR, which were stored in a freezer at -20°C, were not amplified again, through neither a multiplex reaction nor a new singleplex reaction. For the other 17 samples, the gapdh target was not amplified, which could be the result of inefficient DNA extraction or DNA degradation after extraction. Both of these cases should be assumed to yield false-negative results and should not be included in the calculation of infection rates. Among the samples that were negative for both targets, 47.7% (11/21) were derived from skin. Less DNA is usually extracted from skin samples compared with the spleen and liver, and this DNA is likely more susceptible to integrity loss due to DNA degradation.

The testing of two liver samples from two animals [one rodent (*Calomys expulsus*) and one marsupial (*Gracilianus agilis*)] using the multiplex system yielded positive results only for the kDNA target. These samples likely contained a huge amount of *Leishmania* DNA, and the competition between the primer pairs resulted in the amplification of only the *Leishmania* sp. target, because the conditions of the multiplex reaction favor amplification of kDNA over the gapdh gene. Multiplex PCR systems are always subject to competition between the primer sets used and their respective target sequences [9]. Although gapdh amplification was not detected in these two samples (1.9%), this result did not interfere with the main goal of the proposed multiplex system, which is to detect the housekeeping gapdh gene in negative samples, because the viability of DNA in the samples that show *Leishmania* kDNA, amplification is confirmed with detection of the infection.

The multiplex PCR system proposed herein allows for the simultaneous detection of a constitutive mammalian gene (gapdh) and *Leishmania* sp. kDNA in tissue samples derived from distinct wild mammal species. kDNA is the most employed target in the detection of *Leishmania* sp. infection due its high copy number in each cell [10], and we included detection of the mammal gapdh sequence under the conditions established for parasite detection. Our multiplex system constitutes a screening method of the diagnosis of *Leishmania* infection because it eliminates false-negative results. At a subsequent moment, and focusing only on the positives samples from the first assay, identification of the *Leishmania* species can be achieved using appropriate molecular targets [13,23]. In this study, we confirmed the viability of DNA in the samples and, when positive, detected *Leishmania* KDNA in tissue samples from humans, experimentally infected rodents and different species of naturally infected wild mammals. The multiplex system presented herein can be incorporated for routine *Leishmania* sp. molecular diagnosis in mammalian samples.

## Supporting information

S1 Fig Multiple alignment of catalytic gapdh genes from different mammals and *Leishmania* species. The selected genes from *L*. *donovani—*13388249; *L*. *infantum—*5073932 and *L*. *mexicana—*13448275 encode cytosol isoenzymes, whereas the selected genes from *L*. *major—*5653844; *L*. *braziliensis*—5417820; *L*. *tarentolae*—DQ092549; *L*. *panamensis—*22577345; *L*. *chagasi*—KF041811 and *L*. *lainsoni*—KP197180 encode glycosomal isoenzymes. All species were structurally aligned using ClustalW in CLC—Workbench software (version 6), and all sequences were accessed from the NCBI and EMBL-EBI databases using UniProt and InterPro resources. Light grey—catalytic domain for each gapdh sequence; Dark Grey—localization of each primer in the alignment: Forward (ACCACAGTCCATGCCATCAC) and reverse (GTCAGGTCCACCACTGACAC). Bar plot—nucleotide conservation; consensus—sequence generated through the most conserved nucleotides between all aligned sequences.
(TIF)

S1 Table In silico analysis of sequence identity between the gapdh oligonucleotides designed in the present study and representative gapdh sequences of mammals and *Leishmania* species. (DOCX)
