# Supplementary figures and images for: Multiplex PCR as a tool for the diagnosis of Leishmania spp. kDNA and the gapdh housekeeping gene of mammal hosts
Source: PLoS One. 2017 Mar 16;12(3):e0173922. doi: 10.1371/journal.pone.0173922 (PMC5354409; doi:10.1371/journal.pone.0173922)

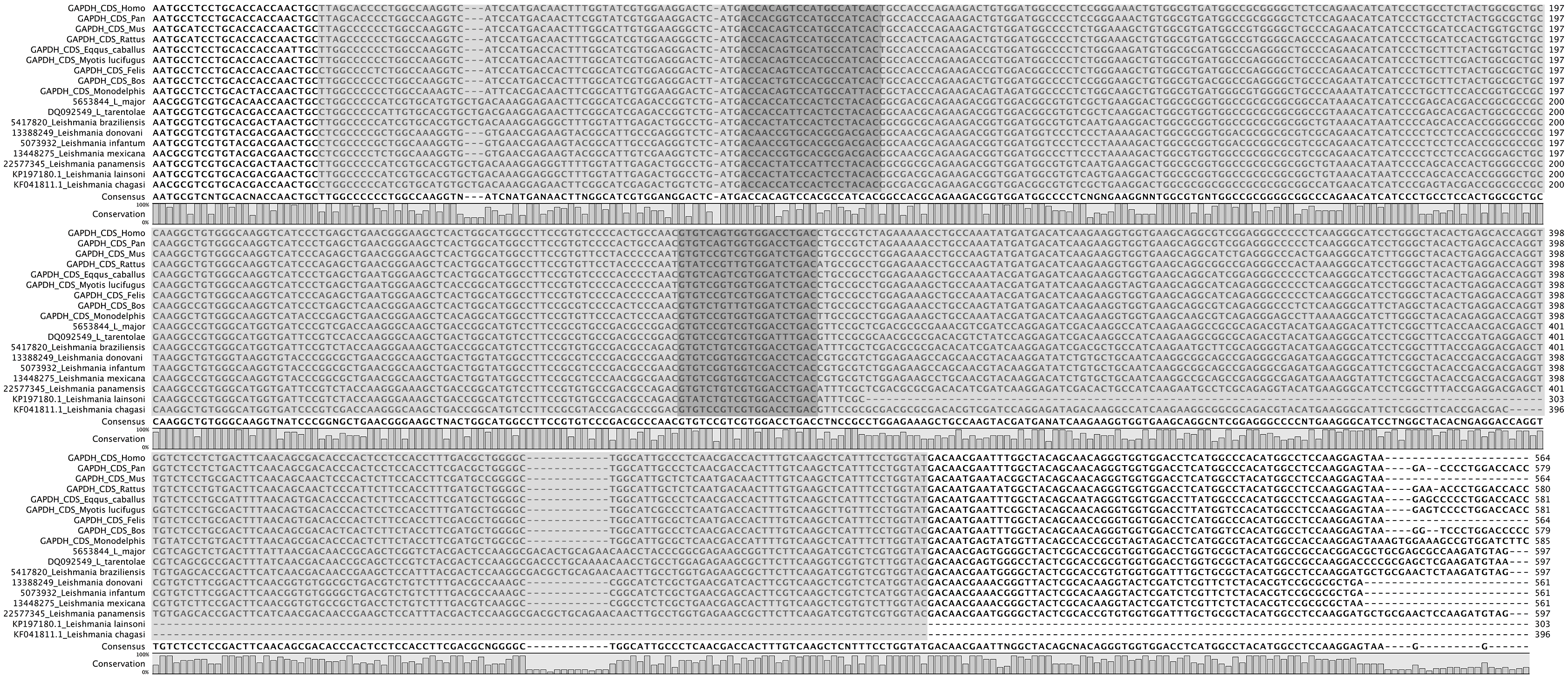

Supplement: S1 Fig — The selected genes from L. donovani—13388249; L. infantum—5073932 and L. mexicana—13448275 encode cytosol isoenzymes, whereas the selected genes from L. major—5653844; L. braziliensis—5417820; L. tarentolae—DQ092549; L. panamensis—22577345; L. chagasi—KF041811 and L. lainsoni—KP197180 encode glycosomal isoenzymes. All species were structurally aligned using ClustalW in CLC—Workbench software (version 6), and all sequences were accessed from the NCBI and EMBL-EBI databases using UniProt and InterPro resources. Light grey—catalytic domain for each gapdh sequence; Dark Grey—localization of each primer in the alignment: Forward (ACCACAGTCCATGCCATCAC) and reverse (GTCAGGTCCACCACTGACAC). Bar plot—nucleotide conservation; consensus—sequence generated through the most conserved nucleotides between all aligned sequences. (TIF) [file pone.0173922.s001.tif]
