# Supplementary material for: Multiplex PCR as a tool for the diagnosis of Leishmania spp. kDNA and the gapdh housekeeping gene of mammal hosts
Source: PLoS One. 2017 Mar 16;12(3):e0173922. doi: 10.1371/journal.pone.0173922 (PMC5354409; doi:10.1371/journal.pone.0173922)
Supplement: S1 Table — (DOCX) [file pone.0173922.s002.docx]

**Table S1.** *In silico* analysis of sequence identity between the gapdh oligonucleotides designed in the present study and representative gapdh sequences of mammals and *Leishmania* species.

|  | **Gene ID** | **Gene location** | **Species** | **Total score** | **Query cover** | **E value** | **Identity** | **Primer** |
| --- | --- | --- | --- | --- | --- | --- | --- | --- |
| 1 | 2597 | NC_000012.12 | *Homo sapiens* | 197 | 100% | 4,00E-08 | 100% | Forward |
|  |  |  |  | 134 | 100% | 4,00E-08 | 100% | Reverse |
| 2 | 451783 | NC_006479.4 | *Pan troglodytes* | 177 | 100% | 1,00E-05 | 95% | Forward |
|  |  |  |  | 134 | 100% | 4,00E-08 | 100% | Reverse |
| 3 | 14433 | NC_000072.6 | *Mus musculus* | 251 | 100% | 5,00E-08 | 100% | Forward |
|  |  |  |  | 124 | 90% | 0,011 | 100% | Reverse |
| 4 | 24383 | NC_005103.4 | *Rattus norvegicus* | 191 | 100% | 4,00E-08 | 100% | Forward |
|  |  |  |  | 81.8 | 80% | 0,01 | 100% | Reverse |
| 5 | 102245543 | NW_005366017.1 | *Myotis brandt* | 198 | 100% | 1,00E-05 | 95% | Forward |
|  |  |  |  | 192 | 100% | 0,003 | 90% | Reverse |
| 6 | 102888425 | NW_006437736.1 | *Pteropus alecto* | 183 | 100% | 1,00E-05 | 95% | Forward |
|  |  |  |  | 155 | 100% | 0,002 | 90% | Reverse |
| 7 | 403755 | NC_006609.3 | *Canis lupus* | 169 | 100% | 1,00E-05 | 95% | Forward |
|  |  |  |  | 79.8 | 70% | 0,009 | 100% | Reverse |
| 8 | 100033897 | NC_009149.2 | *Equus caballus* | 216 | 100% | 1,00E-05 | 95% | Forward |
|  |  |  |  | 177 | 100% | 0,003 | 90% | Reverse |
| 9 | 281181 | AC_000162.1 | *Bos taurus* | 208 | 100% | 0,003 | 90% | Forward |
|  |  |  |  | 210 | 90% | 0.01 | 100% | Reverse |
| 10 | 102422339 | NW_005871058.1 | *Myotis lucifugus* | 149 | 100% | 1,00E-05 | 95% | Forward |
|  |  |  |  | 190 | 100% | 0,003 | 90% | Reverse |
| 11 | 5653844 | NC_007271.2 | *Leishmania major* (strain Friedlin) | 43.1 | 60% | 0.66 | 100% | Forward |
|  |  |  |  | 62.9 | 100% | 7,00E-04 | 90% | Reverse |
| 12 | DQ092549 | DQ092549.1 | *Leishmania tarentolae* | 57.5 | 60% | 0.66 | 100% | Forward |
|  |  |  |  | 24.3 | 69% | 7,00E-04 | 100% | Reverse |
| 13 | 5417820 | NC_009322.2 | *Leishmania braziliensis MHOM/BR/75/M2904* | 45.1 | 60% | 0.17 | 100% | Forward |
|  |  |  |  | 55.0 | 100% | 7,00E-04 | 90% | Reverse |
| 14 | 13388249 | NC_018263.1 | *Leishmania donovani BPK282A1* | 14.4 | 35% | 0.65 | 100% | Forward |
|  |  |  |  | 59.5 | 45% | 0.17 | 100% | Reverse |
| 15 | 5073932 | NC_009420.2 | *Leishmania infantum strain JPCM5* | 14.4 | 35% | 0.65 | 100% | Forward |
|  |  |  |  | 59.5 | 45% | 0.17 | 100% | Reverse |
| 16 | 13448275 | NC_018324.1 | *Leishmania mexicana MHOM/GT/2001/U1103* | No significant similarity found | | | | Forward |
|  |  |  |  | 47.1 | 45% | 0.17 | 100% | Reverse |
| 17 | 22577345 | NC_025877.1 | *Leishmania panamensis strain MHOM/PA/94/PSC-1* | 45.1 | 60% | 0.17 | 100% | Forward |
|  |  |  |  | 24.3 | 100% | 7,00E-04 | 90% | Reverse |
| 18 | KP197180 | KP197180.1 | *Leishmania lainsoni CBT75* | 43.1 | 90% | 0.39 | 100% | Forward |
|  |  |  |  | 69.4 | 100% | 2 | 100% | Reverse |
| 19 | KF041811 | KF041811.1 | *Leishmania chagasi CBT 13* | 43.1 | 60% | 0.45 | 100% | Forward |
|  |  |  |  | 53.0 | 100% | 5,00E-04 | 90% | Reverse |
